# Mechanisms for Chromosome Segregation in Bacteria

**DOI:** 10.3389/fmicb.2021.685687

**Published:** 2021-06-16

**Authors:** Christos Gogou, Aleksandre Japaridze, Cees Dekker

**Affiliations:** Department of Bionanoscience, Kavli Institute of Nanoscience Delft, Delft University of Technology, Delft, Netherlands

**Keywords:** bacterial chromosome, chromosome segregation, entropic segregation, structural maintenance of chromosome, ParABS system, prokaryotic segregation mechanisms

## Abstract

The process of DNA segregation, the redistribution of newly replicated genomic material to daughter cells, is a crucial step in the life cycle of all living systems. Here, we review DNA segregation in bacteria which evolved a variety of mechanisms for partitioning newly replicated DNA. Bacterial species such as *Caulobacter crescentus* and *Bacillus subtilis* contain pushing and pulling mechanisms that exert forces and directionality to mediate the moving of newly synthesized chromosomes to the bacterial poles. Other bacteria such as *Escherichia coli* lack such active segregation systems, yet exhibit a spontaneous de-mixing of chromosomes due to entropic forces as DNA is being replicated under the confinement of the cell wall. Furthermore, we present a synopsis of the main players that contribute to prokaryotic genome segregation. We finish with emphasizing the importance of bottom-up approaches for the investigation of the various factors that contribute to genome segregation.

## Introduction

In all domains of life, proliferation of organisms essentially includes a faithful replication of the genetic material to pass it on to their offspring. The separation of newly copied DNA material into individual physical chromosomes that are spatially relocalized toward the daughter cells is generally called DNA segregation.

**FIGURE 1:**
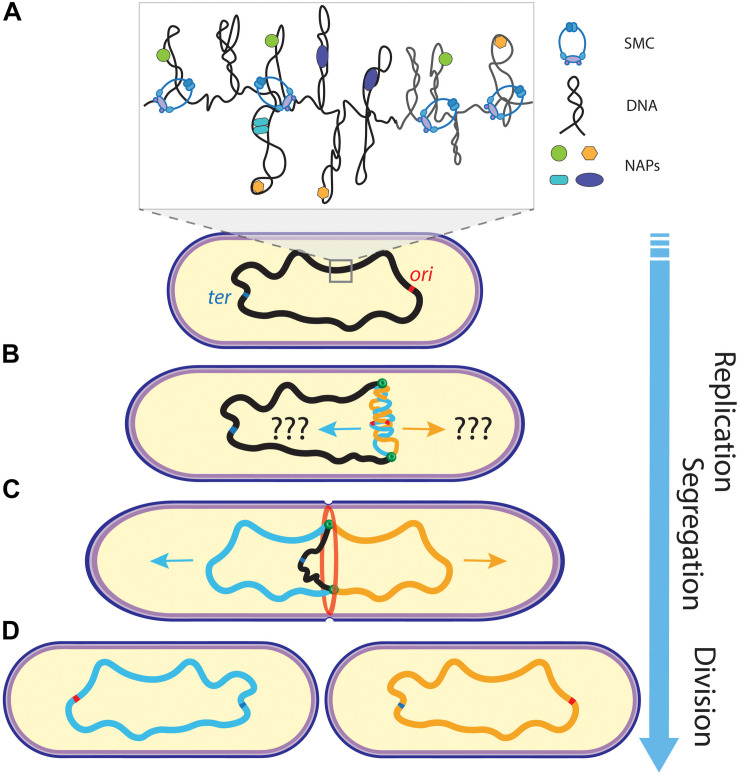
Schematics of chromosome replication and segregation in bacteria. **(A)** A mother chromosome (black) with an origin (ori) and terminus (ter) of replication site resides in the cell. Inset shows the compacted organization of the chromosome into supercoiled domains organized by NAPs and SMCs. **(B)** Upon replication initiation at ori, sister chromosomes (blue and orange) are being synthesized by the replisomes (green dots). **(C)** While the replisomes move bi-directionally along the chromosome arms to further replicate the DNA, the sister chromosomes segregate into opposite cell halves and the cell starts to divide (through the divisome ring shown in red). **(D)** Ultimately, two daughter cells are formed after cell division, with each containing one copy of the chromosome. Arrow of time runs from top to bottom.

Notably, the ∼1–10 megabase pair (Mbp) (Blattner et al., 1997; Kunst et al., 1997; Schoolnik and Yildiz, 2000; Nierman et al., 2001) sized genomes of bacteria need to be highly condensed in order to fit inside the volume of a bacterial cell. Bacteria realize such a strong condensation through DNA supercoiling (Travers and Muskhelishvili, 2005; Dorman and Dorman, 2016), DNA-binding Nucleoid Associated Proteins (NAPs) (Anuchin et al., 2011; Ohniwa et al., 2011; Wang et al., 2011), and other DNA-compacting proteins like the DNA-loop-extruding Structural Maintenance of the Chromosome (SMC) complexes (Lindow et al., 2002; Postow et al., 2004). Replication of the circular prokaryotic chromosome initiates at a dedicated origin of replication (ori) locus and terminates near the terminus of replication (ter) on the opposite side of the chromosome (Nielsen et al., 2006; David et al., 2014; Wang et al., 2014a; Youngren et al., 2014; Cass et al., 2016), while segregation occurs simultaneously with the replication process (Figure 1). In the replication process, the replication machinery (the replisomes) acts bi-directionally: one traversing along each chromosome arm to duplicate the DNA (Japaridze et al., 2020). Throughout the 20–200 min of a typical bacterial cell cycle, the sequential positioning of chromosomal regions is tightly regulated. Obviously, segregation requires temporal coordination with the cell division (Mierzejewska and Jagura-Burdzy, 2012; den Blaauwen, 2013; Adams et al., 2014; Dewachter et al., 2018; Marczynski et al., 2019; Reyes-Lamothe and Sherratt, 2019; Pióro and Jakimowicz, 2020). Divisome constriction needs to be postponed until the segregation is finalized, as failure in doing so will result in “guillotining” of the nucleoid, anucleate cells, or the complete inhibition of the cell division (Mulder and Woldringh, 1989; Woldringh et al., 1990; Åkerlund et al., 2002; Wu and Errington, 2004; Lee and Grossman, 2006; Mierzejewska and Jagura-Burdzy, 2012; den Blaauwen, 2013; Adams et al., 2014; Dewachter et al., 2018).

What are the mechanisms that orchestrate chromosome segregation in prokaryotes that ensure that each daughter cell faithfully acquires its own chromosome copy? In this review, we discuss the various underlying mechanisms for segregation in the best studied model bacterial species *Caulobacter crescentus*, *Bacillus subtilis*, and *Escherichia coli*. Firstly, we address early models that explain segregation as a consequence of being coupled to other cellular growth processes. Secondly, we discuss recent studies of SMCs that organize and compact DNA, and their alleged role in mediating global segregation, as well as the role of topoisomerases that catalyze disentanglement by resolving knots. Thirdly, two well-studied segregation apparatuses are outlined that are known to actively exert pushing or pulling forces on specific sequences of plasmids or chromosomes. Finally, we review the emerging understanding of contributions by entropic de-mixing of DNA polymers as drivers of spontaneous segregation.

Although many of these processes have been studied and reported separately, they do not act independently but jointly co-operate in ensuring reliable DNA segregation. Understanding the coupling between these multiple factors is important to uncover the mysteries of genetic proliferation. The principles of the combinatorial segregation mechanisms are likely not limited to bacteria but also form the basis of similar process in the more complex archaea and eukaryotes.

## Early Models

**FIGURE 2:**
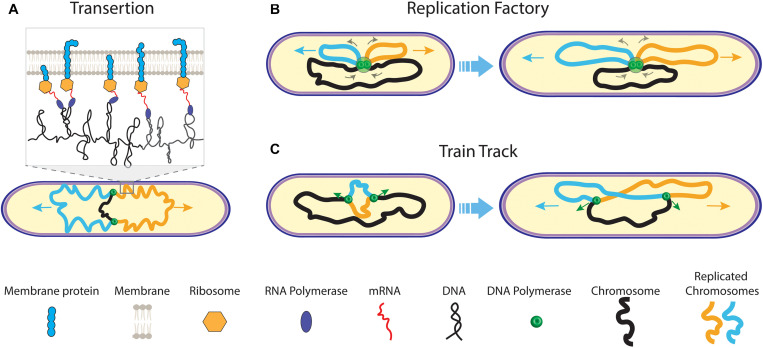
Early models of prokaryotic DNA segregation. **(A)** The transertion model. During replication of the mother chromosome (black) by the replisomes (green dots), co-translational transcription of membrane protein mRNA leads to tethering of the sister chromosomes (orange and blue) to the cell membrane. As genes on one chromosome are biased in their position relative to genes on the other chromosome, a transertion area forms on the cell membrane for each sister (inset). Both transertion areas move in opposite direction on the elongating cell membrane. **(B)** The replication factory model. During replication, centrally fixed replisomes extrude replicated sister chromosomes (orange and blue) toward the cell pole, concomitantly pulling the yet un-replicated mother chromosome (black) inward. **(C)** The train track replication model. The two replisomes independently act on and move along the separate chromosome arms during replication. Their position in the cell is determined by the localization of the to-be copied genetic material.

The symmetric distribution of copied genomes into opposite cell halves was the subject of different models that coupled segregation to other processes such as cell growth and replication. Very early on, Jacob et al. (1963) postulated segregation as being governed by the attachment of DNA to the bi-directionally elongating cell wall. Based on this cell wall anchoring, Kleppe et al. (1979) proposed the so-called transertion model which was further developed in parallel by Norris (1995) and Woldringh et al. (1995); Woldringh (2002)). According to this model, the bacterial nucleoid is organized into supercoiled segments, and this nucleoid is separated from the cytoplasm through volume exclusion resulting from crowding interactions. The model emphasizes the translation of membrane proteins that is occurring co−transcriptionally, i.e., translation of the protein occurs simultaneously with the transcription of genes (Woldringh et al., 1995). Genes coding for membrane proteins will therefore become transiently bound to the membrane (Figure 2A). As the result, nearby genes expressed on the same chromosome also get localized to that spot near the membrane. Upon DNA replication, genes on the two daughter chromosomes will compete with each other for membrane binding which is self-enhanced upon expression of new genes (Roggiani and Goulian, 2015), leading to the formation of separate transertion areas per chromosome. Although anchoring of plasmids (Lynch and Wang, 1993) and chromosome regions to the membrane has been observed in some bacterial species (Leibowitz and Schaechter, 1975), there is, however, no clear evidence that the transertion plays a major role in the chromosome segregation.

Chromosome replication and segregation occur simultaneously in bacteria. Another early segregation mechanism involved the coupling of both these processes, where fluorescence microscopy data by Lemon and Grossman (1998) appeared to indicate a fixed replisome near the cell center in *B. Subtilis.* A central anchoring of the replisome could allow the cell to push the newly synthesized chromosome sisters bi-directionally outward (Figure 2B). This is known as the “replication factory model”. Fluorescently tagged genomic foci were moving toward the replisome at mid cell before duplication, suggesting that the DNA is actively pulled inward by the replisome before being extruded outward in the opposite directions again (Lemon and Grossman, 2000). While similar observations were reported for *E. coli* (Mangiameli et al., 2017), conflicting findings were also reported for both *B. subtilis* (Migocki et al., 2004) and *E. coli* (Reyes-Lamothe et al., 2008), where the replisomes were not fixed relative to mid cell in live cells but rather moved along the chromosome in accordance with a “train track model” (Figure 2C). Fluorescence time-lapse imaging data revealed that the replisome foci for both organisms would split into two–one focus for each replisome that replicates a separate chromosome arm (Japaridze et al., 2020). In widened cells, we similarly visualized that replisomes assembled near ori before splitting to move separately over opposing chromosome arms. For *C. crescentus* (Jensen et al., 2001), movement of the replication machinery was also observed throughout replication. The replisome movement in *C. crescentus*, *E. coli*, and *B. subtilis*, as well as the splitting of replisome foci in the latter two organisms strongly argue against the factory model. The causation between replication and segregation may even be inverted: A central replisome position may be a consequence of the newly synthesized sister chromosome moving outward while the mother chromosome moves inward. Another early model by Kleckner et al. (2014) suggested that segregating forces result from the build-up of the mechanical stress by the chromosome replication, where segregation would result from stress relaxation upon the loss of sister cohesion (Bates and Kleckner, 2005; Javer et al., 2014; Lim et al., 2014).

## SMCs Compact Sister Chromosomes Into Individual Entities

**FIGURE 3:**
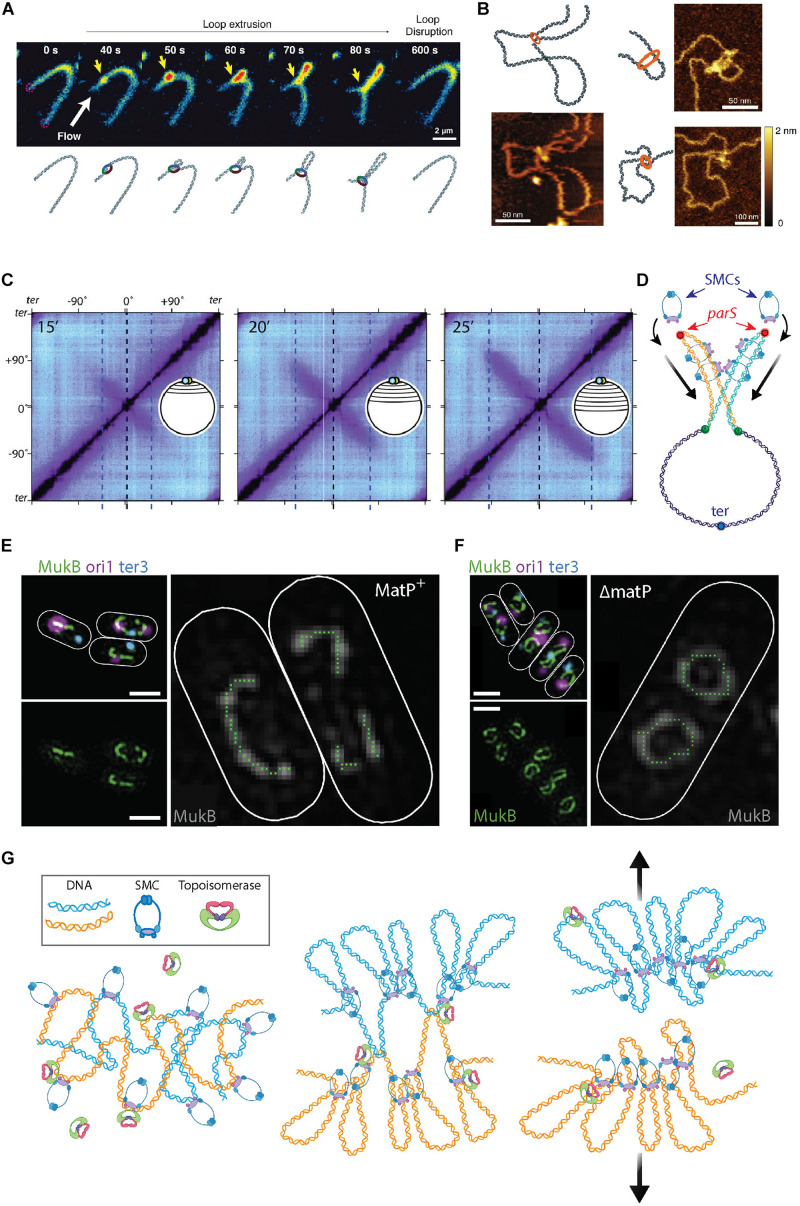
The roles of SMCs in organizing and segregating the bacterial chromosomes. **(A)** Time-lapse images of a single SMC complex (yellow arrow) that extrudes a DNA loop *in vitro*. From Ganji et al. (2018). **(B)** Atomic force microscopy images of a single SMC complex bound to the stem of a DNA loop of varying sizes. From Ryu et al. (2020). **(C)** Hi-C contact maps show the progressive juxtaposition of chromosome arms as the downward-oriented diagonal lines that increase in size over time. From Wang et al. (2017). **(D)** Schematic representation of SMC molecules loading onto the *parS* sites (red dots) at newly replicated sister DNA (orange and blue strands) from the mother (purple strand). Upon loading, the SMC complexes slide over the DNA toward the terminus of replication (blue focus), juxtaposing the chromosome arms of the two sister chromosomes. **(E)** Fluorescent microscopy images of the MukB SMC (green) structures in *Escherichia coli* cells. The smaller images on the left show an overlay of MukB with the origin (magenta) and terminus-of-replication (blue) sites. The large image shows a trace of the MukB signal, which visualizes the horse-shoe-shaped half ring, that is discontinued at ter by the presence of MatP. Scale bars 1 μm. From Mäkelä and Sherratt (2020). **(F)** Same as panel **(E)** but for cells where MatP was deleted. Here, the horse-shoe shapes are closed into a fully circular structure. From Mäkelä and Sherratt (2020). **(G)** *Left* Sketch of two entangled sister chromosomes directly after replication. *Middle* Chromosome compaction by the action of SMCs. DNA loop extrusion by SMCs leads to a bottle brush chromosome structure, but full segregation between sister chromosomes is impeded by topological links between the sister chromosomes. *Right* Concatenations between the chromosome sister are resolved by topoisomerase action and entropic repulsion completes the segregation.

Structural Maintenance of the Chromosome are an important class of proteins that organize genomes in all domains of life (Cobbe and Heck, 2004). Indeed, all bacteria have such SMCs (Hirano, 2016) that are loaded onto the genome, for example near the ori regions in *B. subtilis* (Wang et al., 2014b) and *C. crescentus* (Tran et al., 2017). These complexes densely compact the chromosome by locally looping DNA. Although not demonstrated yet for bacterial SMCs, *in vitro* visualization of the structurally similar eukaryotic condensin SMC showed that these SMCs are capable of tethering to the DNA and utilizing ATP hydrolysis to extrude DNA loops (Ganji et al., 2018) (Figure 3A). Similarly, AFM imaging captured DNA loops of varying size with a single SMC complex of budding yeast at the base of the loop (Figure 3B). These SMCs exhibited two predominant conformations, (Ryu et al., 2020) indicating that the SMCs undergo very sizable conformational changes to progressively extrude DNA. Here, we describe the compacting functions of SMCs as well as emergent insights in the role they play in organizing replicated DNA into individual sister chromosomes in anticipation of their subsequent segregation.

The major SMC in *B. subtilis* is called the BsSMC condensin and it is associated with compaction of the chromosome (Kleine Borgmann et al., 2013). Recruitment of condensin is mediated through interactions with ParB (Gruber and Errington, 2009) proteins that bind to ori-proximal *parS* sequences. Endogenous expression of a chemically degradable version of the SMC protein elucidated that ParB-dependent SMC recruitment is essential in fast growing bacteria (Wang et al., 2014b). Using high-throughput chromosome conformation capture (Hi-C) techniques, Rudner et al. showed how different genetic loci spatiotemporally relate to each other (Wang et al., 2017, 2018). The Hi-C method characterizes chromosome folding by measuring the rate of interactions between genomic loci that are nearby in space but may be separated by a large distance genomically (for reviews, see de Wit and de Laat, 2012; Denker and De Laat, 2016; McCord et al., 2020). Population-based Hi-C at different stages of segregation provided evidence that, following ParB-mediated loading onto DNA, SMCs “zip” along a single chromosome from the origin to the terminus of replication, while holding on to both chromosome arms and thus sequentially aligning regions on the opposing arms (Wang et al., 2017, 2018). This is indicated by an appearance, increasing in size over time, of a second diagonal that is perpendicular to the main one (Figure 3C). Using fluorescent microscopy, it was observed that GFP-tagged SMCs nucleate at *parS* before spreading out over more distal regions. The data indicate that multiple copies of SMCs consecutively bind at, and slide away from, *parS* during the observed juxtaposition of chromosome arms. Similar SMC behavior was recently observed in *C. crescentus* (Tran et al., 2017) cells. Furthermore, Karaboja et al. (2021) recently showed that in *B. subtilis* these processive SMCs ultimately unload near the ter macrodomain. It was demonstrated that BsSMC is also capable of entrapping DNA within its structure (Wilhelm et al., 2015). The entrapment and sliding suggest a mechanism for chromosome segregation where BsSMCs are loaded onto each replicating sister chromosome whereupon they impose individualization as the complexes slide over the DNA. This individualization self-organizes a segregation of the daughter nucleoids, see Figure 3D.

In *E. coli*, the major SMC is the tripartite MukBEF complex that comprises two copies each of MukB, MukE, and MukF (Valentin et al., 2014). Hi-C data revealed a loss of long-ranged (scales > 280 kb) intra-chromosomal contacts *in vivo* upon MukBEF deletion (Lioy et al., 2018), suggesting that MukBEF is organizing chromosomal loops of hundreds of kb in size in the *E. coli* nucleoid. MatP protein, a protein that specifically binds *matS* sites in the ter region (Mercier et al., 2008), was found to prevent MukBEF-induced long-range contacts in the ter macrodomain (Lioy et al., 2018). Recent *in vivo* 3D SIM imaging revealed that, upon sixfold upregulation of MukBEF, the proteins formed a horse-shoe-like backbone structure, that co-aligned with the chromosome structure, from which DNA loops were inferred to emanate (Mäkelä and Sherratt, 2020) (Figure 3E). Such a MukBEF backbone of the chromosome did not form at the ter region in the presence of the MatP protein, consistent with the antagonistic action of MatP on MukBEF (Nolivos et al., 2016). Deletion of MatP led to the closing of the MukBEF ring through ter (Figure 3F), resulting in global re-orientation and re-positioning.

A prominent difference between the DNA-binding mechanisms of SMCs in *B. subtilis* and *C. crescentus*, versus those in *E. coli*, is that the latter lacks *parS* sites on its genome for the loading of SMCs near ori (Livny et al., 2007). A recent simulation study, however, showed that some form of preferential loading is nevertheless needed to account for the experimental observations of ori and MukBEF dynamics (Sherratt et al., 2019). Although no mechanism was so far identified for the loading of MukBEF onto the *E. coli* chromosome, a role might be ascribed to the MatP protein which drives MukBEF away from the ter region (Nolivos et al., 2016) which leads to a gradient of MukBEF along the chromosome. Simulations showed that, due to compaction and looping of DNA by MukBEF, preferential loading of MukBEF near ori would cause the formation of a MukBEF focus with ori at the cell centre (Murray and Sourjik, 2017; Sherratt et al., 2019). Cell elongation resulted in splitting of these MukBEF foci to the cell quarter positions, and subsequently a segregation of duplicated oris toward these MukBEF foci. Another player in regulating the distribution of the SMCs along the genome is the XerCD/dif system, where XerC and XerD proteins bind at the dif site in the terminus domain and catalyze the resolution of chromosome dimers that arise as a result of replication (Blakely et al., 1993; Cornet et al., 1997; Sciochetti et al., 1999, 2001; Lesterlin et al., 2004; Midonet and Barre, 2015). It was recently shown that XerD functions as a site-specific unloader of SMC complexes in *B. subtillis* (Karaboja et al., 2021), although such mechanism has not been shown yet in *E. coli*.

Structural maintenance of the chromosome play a regulatory role in coordinating chromosome segregation and cell division. Deletion of the *E. coli* MukB or deletion of the *B. subtilis* SMC, results in guillotining of the nucleoid as well as anucleation of the cells (Niki et al., 1991; Moriya et al., 1998). In *E. coli*, the MatP protein, which binds to the ter region of the chromosome and prevents MukB from binding it, directly connects to the ZapB and ZapA proteins in the divisome. These three proteins interact to form a complex that anchors the ter region to the Z-ring (Espéli et al., 2012; Männik et al., 2016), thus orchestrating divisome positioning with chromosome segregation. A similar coupling of the terminus and the divisome was recently also found in *C. crescentus* (Ozaki et al., 2020, 2021). ZapA and ZauP, the functional counterparts of ZapA and ZapB in *E. coli*, interact with ZapT (the MatP counterpart). Deletion of the ZapT protein resulted in delayed cell division and altered divisome localization, indicating that this mechanism of chromosome anchoring to the divisome could be a general mechanism of coupling chromosome segregation and division.

Structural maintenance of the chromosomes also interact with another important player in chromosome organization and segregation–the bacterial topoisomerases (TopoIV) that can resolve knots and supercoiling. For both *B. subtilis* and *E. coli*, the mutual interaction between their respective SMC and TopoIV has been well described, especially for the latter (Lindow et al., 2002; Tadesse and Graumann, 2006; Nicolas et al., 2014; Wang et al., 2014b; Kumar et al., 2017). MukBEF recruits TopoIV to the chromosome and thus stimulates the relaxation of negative supercoils (Hayama and Marians, 2010; Li et al., 2010; Hayama et al., 2013; Vos et al., 2013; Nicolas et al., 2014). TopoIV is capable of relieving supercoiling stress through the sequential breaking, passing, and re-ligation of the double-stranded DNA, thereby reducing the linking number of the chromosome (Wang, 1998; Crisona et al., 2000; Seol et al., 2013; Ashley et al., 2017). This strand-passage activity is crucial for the detachment of topologically catenated sister chromosomes after termination of replication (Zechiedrich and Cozzarelli, 1995; Zechiedrich et al., 1997; Seol et al., 2013). Orlandini et al. (2019) simulated how condensins could slide over an entangled ring polymer to sequester the knots. Building on this process, these authors (Orlandini et al., 2019) and others (Goloborodko et al., 2016; Brahmachari and Marko, 2019) showed how the inter- and intra-chromosomal linkage of the polymers substrates was resolved through strand-passage activity of topoisomerases (TopoII) bound to these loop-extruding SMCs.

In eukaryotes, simulations similarly demonstrated that loop extrusion combined with such topoisomerase action resulted in the formation of compacted chromosomes (Goloborodko et al., 2016) consisting of an axial superstructure of condensins with DNA loops that are peripherally protruding, resembling a “bottle brush” structure (Goloborodko et al., 2016; Brahmachari and Marko, 2019). This sufficed to spontaneously segregate two highly entangled and interconnected DNA polymers into separate compacted structures–both for eukaryotic and prokaryotic chromosomes (Brahmachari and Marko, 2019) (Figure 3G).

## Pulling and Pushing Plasmids and Chromosomes

Multiple protein apparatuses have been identified that actively push or pull plasmids or sister chromosomes apart. Plasmids are typically much smaller than chromosomes found in bacteria (Shintani et al., 2015), with sizes of ∼1–1,000 kbp versus ∼1–10 Mbp (Shintani et al., 2015), respectively. We first discuss the simpler and more thoroughly studied segregation mechanisms in plasmids. High-copy-number plasmids typically segregate to daughter cells by random Brownian motion and thus do not require elaborate segregation mechanisms (Summers, 1998). By contrast, for low-copy-number plasmids, two types of active partitioning mechanisms have been described in multiple bacterial species whereby plasmids are symmetrically segregated to the opposing cell halves. These are the actin-like *parABS* and *parMRC* systems, that each are comprised of three components: *parS* and *parC* ori-proximal DNA sequences on the plasmid, ParA and ParM motor proteins that provide kinetic energy under hydrolysis of ATP, and ParB and ParR proteins that bind the *parS/C* DNA sequences and connect them to the motor proteins in the systems (Garner et al., 2007; Havey et al., 2012). Due to the relevance for prokaryotic chromosome segregation, this review focusses on these two well-studied partitioning systems rather than on other plasmid segregation mechanisms such as plasmid accumulation at cell poles and plasmid clustering (Million-Weaver and Camps, 2014).

**FIGURE 4:**
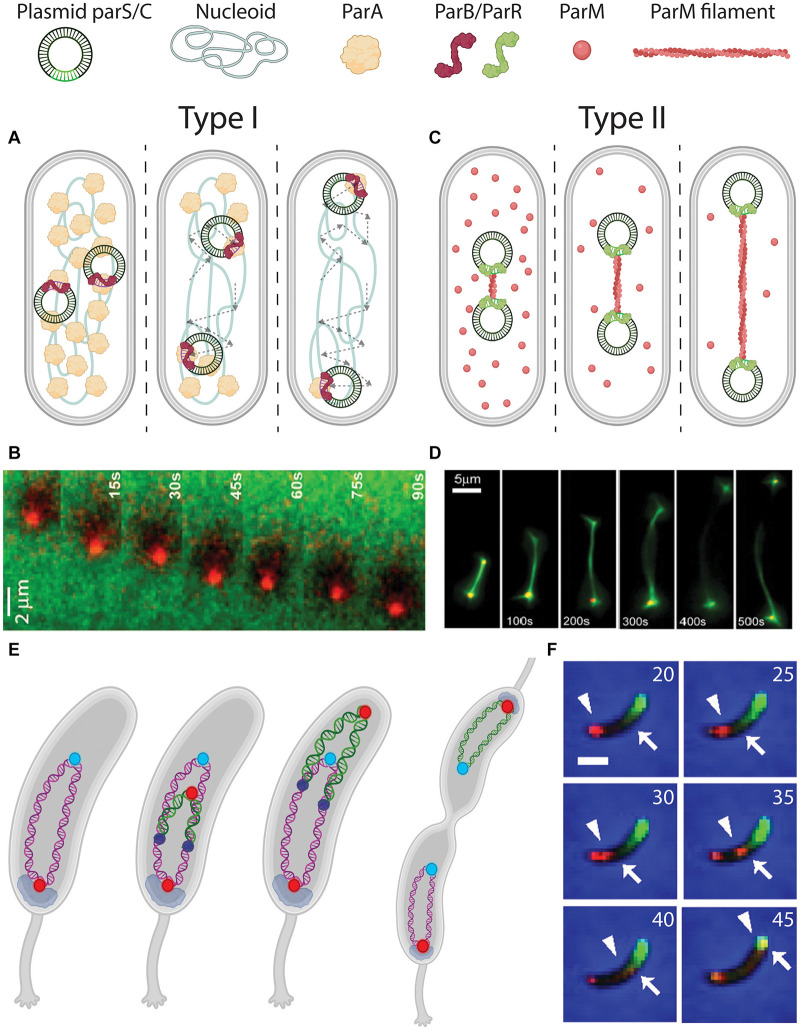
DNA segregation by pushing and pulling. **(A)** Pulling mechanism for plasmid segregation by parABS (Type I). *parS* sequences on the plasmids are bound by ParB (red signal and arrowhead). The ParB-*parS* nucleoprotein then moves outward, with its attached plasmid, through interactions with ParA (green signal and arrow) that is localized between ParB-*parS* and the poles. Dashed arrows indicate the traversed path of the plasmids. **(B)** Fluorescence time-lapse imaging of ParB (red) moving over a ParA carpet (green). From Vecchiarelli et al. (2014). **(C)** Pushing mechanism for plasmid segregation by ParMRC (Type II). *parC* sequences on the plasmids are bound by ParR (green). In between the ParR-*parC* nucleoprotein of a pair of plasmids, a ParM (red) filament polymerizes from soluble monomers, to push the plasmids apart toward the poles. **(D)** Fluorescence time-lapse imaging revealing the *in vitro* growing ParM filament between a pair of plasmids. From Garner et al. (2007). **(E)** Chromosome segregation in *Caulobacter crescentus* bacteria. Before replication, ori (red) is anchored at the old pole by PopZ (gray). After replication initiation (replisomes shown as dark blue circles), one sister ori is pulled over the chromosome toward the new pole. **(F)** Fluorescence time-lapse imaging of parABS-mediated chromosome segregation in *C. crescentus* coexpressing mCherry-ParB (red) and GFP-ParA (green). The ori-proximal ParB that is initially localized at the old pole (indicated by an arrowhead) is duplicated and moves over the ParA gradient (edge indicated by an arrow), depleting it in the process. Scale bar 1μm. Time is indicated in minutes. From Shebelut et al. (2010).

The tripartite *parABS* system (Type I) actively partitions plasmids by a so-called Walker A-type mechanism (Figure 4A). Here, p*arS* sequences are bound by ParB which drags the attached DNA over a carpet of ParA that covers the nucleoid. Fluorescence imaging showed that ParA occupies the nucleoid between ParB-*parS* foci on plasmids and the cell poles (Ringgaard et al., 2009). Upon movement of the ParB-*parS* nucleoproteins over this carpet, the ParA signal depletes. Such observations led to a search for an active filament-based pulling system toward the cell poles. Despite evidence of polymerizing capabilities of ParA *in vitro* (Leonard et al., 2005; Ebersbach et al., 2006), no such filaments were found *in vivo*. Instead, ParA was shown to non-specifically bind chromosomal DNA, which appears to be a necessary step in rendering its interaction with ParB (Volante and Alonso, 2015). Recent studies revealed that ParB binds and hydrolyses CTP in a parS-dependent manner, which in turn is essential for the ParB-ParA interaction (Osorio-Valeriano et al., 2019; Soh et al., 2019; Jalal et al., 2020). *In vitro* reconstitution of the three components showed that ParB locally depletes ParA and then moves up the locally induced gradient, continually depleting proximal ParA (Vecchiarelli et al., 2014) (Figure 4B). This “surfing” of ParB over a ParA gradient leaves a wake of unoccupied DNA without ParA behind it. Computer simulations of this Brownian ratchet model (Hu et al., 2017) could re-capture the biased-random walk plasmid trajectories observed in experiments. This mechanism is capable of successfully segregating plasmids by practically dragging them over a carpet of nucleoid-bound ParA.

The ParMRC segregation system (Type II) partitions the plasmids by forming filaments in-between them that are pushing them apart toward the two poles (Figure 4C). Fluorescence time-lapse imaging in *E. coli* cells showed that pairs of F-plasmids get partitioned to opposing cell halves by an accumulation of ParM signal in between (Campbell and Mullins, 2007). ParR dimers stably bind to tandem-repeated *parC* sequences (Schumacher et al., 2007), forming a nucleoprotein complex that connects to growing ParM filaments that apply a force on the plasmids due to the their growth (Garner et al., 2007). ATP-bound ParM monomers polymerize to form these filaments, and after pushing the plasmids to opposite cell poles, the monomer-bound ATP is hydrolyzed upon which the filaments disassemble (Gayathri et al., 2012). *In vitro* reconstitution of purified ParMRC from *E. coli’s* R1 plasmid (Figure 4D) with ATP showed that these filaments could push pairs of beads over distances as large as 120 μm (Garner et al., 2007).

The *parABS* systems have historically been best studied for their role in plasmid partitioning. Interestingly, evidence has also been presented that the *parABS* plays an important role in the organization and segregation of chromosomes. A genome-wide study showed that almost 70% of 400 investigated prokaryotic species possess chromosomal *parS* sites (Livny et al., 2007). About 75% of those species harbor these loci within 5% of the genomic distance from ori, hinting toward the relevance of *parABS* system for ori segregation in the cell cycle. These species include *C. crescentus*, *Vibrio cholerae*, and *B. subtilis*, while *E. coli* lacks chromosomal *parS* sites despite the role of *parABS* in its plasmid segregation. For *C. crescentus* and *V. cholerae*, *parABS* is indispensable for proper chromosome segregation, as deletion of any of the constituents leads to severe chromosome organization and segregation defects (Mohl and Gober, 1997; Mohl et al., 2001; Yamaichi et al., 2007; Toro et al., 2008; Kadoya et al., 2011).

*Caulobacter crescentus* has its origin of replication and proximal *parS* site held in place at one of its cell poles, known as the old pole (Shebelut et al., 2010) (Figure 4E). Consequently, the rest of the chromosome resides along the long axis of the cell (Viollier et al., 2004). Genomic relocation of *parS* from its native ori-proximal site led to a global reorientation of the entire chromosome (Umbarger et al., 2011). This indicates that forces acting on *parS* are capable of reorienting the entire chromosome. A similar phenotype with a genomic translocation of the primary chromosome *parS* site was observed in *V. cholera* (David et al., 2014).

Upon the initial duplication of ori during replication in *C. crescentus*, the spatial fate of each ori daughter appears determined: One remains stationary at the old pole [anchored by pole organizing protein PopZ (Bowman et al., 2010)], while the other is pulled to the new pole by the *parABS* system (Figure 4F). It was shown that other chromosomal loci follow the trajectory of the latter ori, and consequently the chronological order in which they do so matches their respective genomic distance from ori (Viollier et al., 2004). As for the mechanism for the motion of the newly synthesized ori, akin to the process with plasmids, *parS*-bound ParB processively surfs over a readily present ParA carpet, depleting ParA along its trajectory (Shebelut et al., 2010; Lim et al., 2014). High-resolution fluorescence imaging of GFP-tagged ParB-*parS* complexes showed this motion to be not random (Lim et al., 2014), but of a directed diffusive type. At a later point during the replication cycle, the pulled sister chromosome “flips over” along its longitudinal axis, making the entire segregation resemble the “peeling of a banana skin” (Figure 4E).

*Bacillus subtilis* possesses very closely related versions of ParA (Soj), ParB (SpoOJ), and *parS* on the chromosome (Mysliwiec et al., 1991; Ireton et al., 1994; Funnell, 2016). SIM microscopy revealed an ATP-bound ParA gradient in 3D, where ParA was biased toward co-localizing with high-density regions of DNA throughout the cell cycle (Le Gall et al., 2016). However, the *parABS* system seemed not be vital for proper chromosome segregation for *B. subtilis*, since deletions of ParA or ParB in *B. subtilis* showed that the cells remained capable of partitioning the chromosome, albeit with an untimely ori segregation and an increased rate of replication initiation (Lee and Grossman, 2006).

In *C. crescentus*, ParABS has an additional role of regulating the progression of cell division during segregation (Mierzejewska and Jagura-Burdzy, 2012; den Blaauwen, 2013; Marczynski et al., 2019; Pióro and Jakimowicz, 2020). As ParB-*parS* traverses the cell, the slightly higher ParA concentrations at the new pole stimulate PopZ polymerization into a liquid phase-condensate (Bowman et al., 2008; Ebersbach et al., 2008; Laloux and Jacobs-wagner, 2013) to which ParB-*parS* anchors (Bowman et al., 2008, 2010; Ebersbach et al., 2008; Ptacin et al., 2010), while PopZ reciprocally also promotes ParA’s ATP binding to further increase ParA levels at the poles. As such, PopZ provides a ParA gradient for ParABS segregating action as well as stable ParB-*parS* polar anchoring, therefore positioning sister chromosomes away from the cell center and thus prevents potential nucleoid occlusion during cell division (Mulder and Woldringh, 1989; Woldringh et al., 1990; den Blaauwen, 2013; Adams et al., 2014). Thanbichler and Shapiro (2006) additionally revealed complex formation between ParB and a newly identified protein, MipZ, (Mera et al., 2014) that was shown to directly interfere with the polymerization of FtsZ that is indispensable in forming the Z ring. Through MipZ, the ParB patterns therefore indirectly prevent Z ring constriction as they traverse the cell during segregation, while allowing divisome assembly when being anchored at the cell poles by PopZ. Other proteins, Noc in *B. subtlis* (Wu and Errington, 2004; Adams et al., 2014) and SlmA in *E. coli* (Bernhardt and De Boer, 2005), also interfere with divisome formation by binding DNA anti-correlated with the ter domains, thus further ensuring that constriction takes place when the ter regions are localized at mid cell, i.e., at the end of the segregation stage in dividing cells (Bernhardt and De Boer, 2005; Adams et al., 2014; Misra et al., 2018; Wang et al., 2020).

## Entropy as a Segregating Force

Above we discussed various active biological protein systems such as SMCs and Par systems that globally organize and drive sister chromosomes apart. In recent years, a purely physics-based mechanism has emerged that has won some popularity in explaining chromosomal segregation (Jun and Mulder, 2006; Jun and Wrigth, 2010; Kleckner et al., 2014). This concerns the spontaneous segregation of two intermingled DNA polymers from a mixture. While such a spontaneous de-mixing of two polymers may be counterintuitive, a homogenous mixing of two polymers was found to be entropically unfavorable when confined to a cylindrical cell volume (Jun and Mulder, 2006). Directly after active replication, a mixed state of the DNA sister polymers will limit the number of possible adoptable configurations for each of the two polymers. As a result, the two sister DNAs will spontaneously segregate to maximize entropy. This is predicted to occur under specific conditions such as high initial polymer densities and certain geometries, e.g., a cylindrical confinement as opposed to a spherical confinement.

**FIGURE 5:**
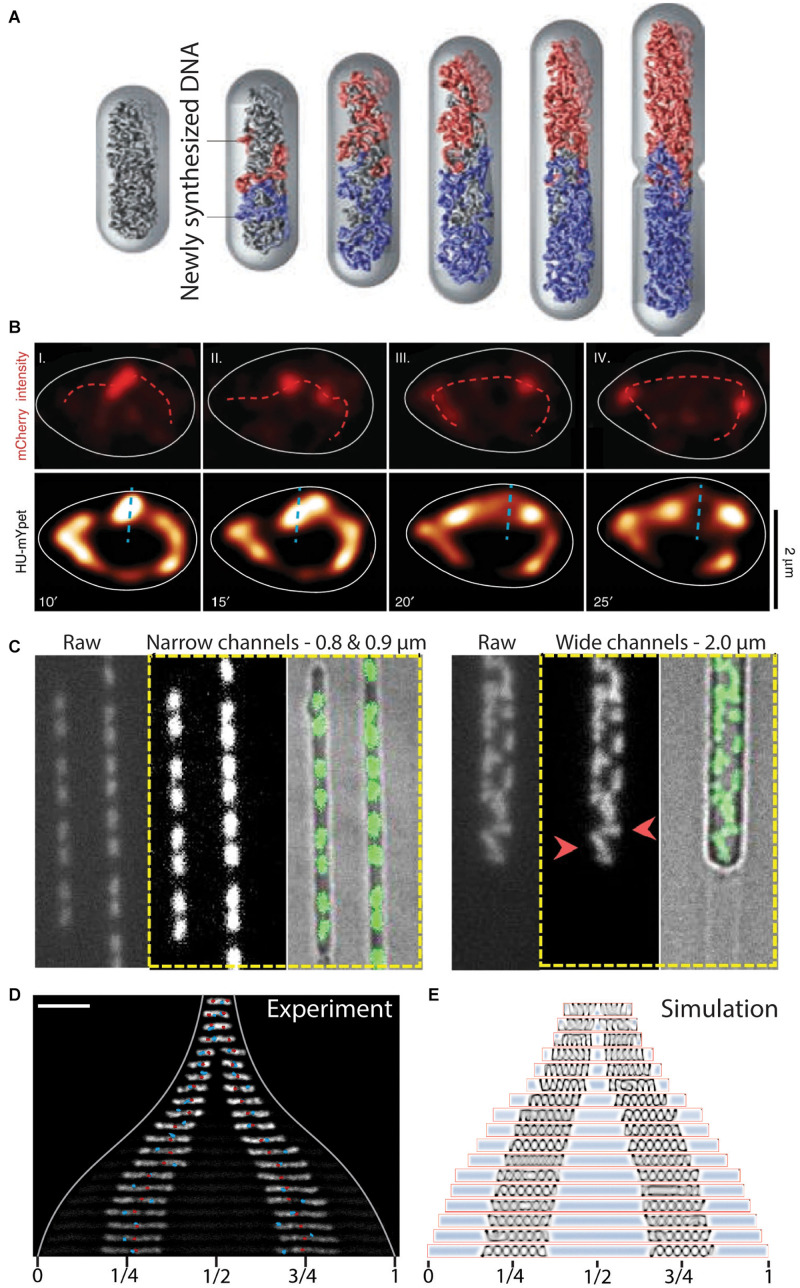
The role of entropy in segregation of chromosomes. **(A)** Simulation of chromosome segregation during replication. Newly synthesized sister chromosomes (blue and red) spontaneously move toward the poles due to entropic forces between the polymers under the confinement of the cell wall. From Jun and Wrigth (2010). **(B)** Independently moving replisomes (labeled in red with mCherry, top row) in widened *E. coli* cells. Initial segregation of the chromosome (labeled in yellow with HU-mYpet, bottom row) is along the short axis of the cell (dashed blue line), indicating a disturbed early ori segregation due to the loss of cell wall confinement on the replicating chromosomes. Time is indicated in minutes. From Japaridze et al. (2020). **(C)** L-form *B. subtilis* cells in microfluidic channels of varying width. Polar segregation of replicating chromosomes is maintained in narrow channels (0.8–0.9 μm), whereas replicated chromosomes are more randomly distributed in the cells in wider channels (2.0 μm). Red arrowheads point at examples of orthogonally and perpendicularly oriented nucleoids relative to the cellular long axis. From Wu et al. (2020). **(D)** Re-distribution of two chromosomes in an elongated *E. coli cell*. The chromosomes increase in size and spontaneously localize at 1/4 and 3/4 positions along the cell length. Ori and ter sites are visualized as red and blue foci, respectively. Scale bar 5 μm. From Wu et al. (2019b). **(E)** Molecular dynamics simulations capture the size and positioning of two chromosomes over the cellular space as a consequence of cytosolic molecular crowding and the entropic spring-like nature of the nucleoids. From Wu et al. (2019b).

Various computational efforts have been made to probe how such an entropic repulsion of DNA polymers may facilitate segregation of different chromosomes within the cellular confinement (Jun and Mulder, 2006; Jung et al., 2012). Jun and Mulder seminally showed how distinct sister chromosomes, or separate chromosome arms, spontaneously de-mix under strong confinement by the cell wall. The de-mixing resulted in the movement of the sister chromosomes to the freely available outer volumes, i.e., toward the cell poles (Jun and Mulder, 2006) (Figure 5A). Consequently, the mother chromosome–still in the process of replication–was kept near the cell center. This phenomenologically captures the sequential segregation of many bacterial genomes. Jun and Wrigth (2010) formulated entropic segregation as dependent on the total polymer length relative to the confinement radius. Plasmids, for example, simply diffuse through a cell since their size is orders of magnitude smaller in size compared to the chromosome. Chromosomes, however, would spontaneously segregate in the typical cylindrical geometries of bacteria.

A recent computational study visualized how successful entropic segregation depends on the relative sizes of the sister chromosomes and the diameter of the cylindrical cell (Polson and Zhu, 2021). Multiple computational efforts on the effect of SMCs in chromosomes also included entropic forces between DNA polymers of sister chromosomes as contributing factors to segregation. For example, Brahmachari and Marko (2019) simulated the repulsion between DNA polymers that emanate from their separate SMC cores as a contributor to chromosome segregation. Sherratt et al. (2019) demonstrated that short-ranged repulsive forces between newly replicated ori regions can lead sister foci to spontaneously end up at opposite cell quarter positions in simulations of *E. coli* segregation. Similarly, El Najjar et al. (2020) modeled the linear movement of ori-proximal loci in *B. subtilis* as consequence from entropic repulsion between newly replicated polymers.

Some experimental evidence for entropy as a driver of chromosome segregation has been obtained, although most evidence concerns indirect indications. The cylindrical confinement of bacteria appears to be of importance since increasing the width of *E. coli* cells in microfluidic channels led to a decreased division rate (Liang et al., 2020). Similarly, loss of cell wall confinement after drug-induced expansion in *E. coli* cells led to a decreased success of timely segregation (Japaridze et al., 2020). Furthermore, the initial segregation of ori in these expanded cells (Japaridze et al., 2020) oriented randomly until the replication of chromosomal mass recovered a level of confinement needed to direct the oris toward the cell poles (Figure 5B). Other studies showed that cell-wall-less cells (so-called L-form cells) exhibited typical segregation defects, such as more randomly oriented nucleoids that physically separated from one another only rarely, while successful segregation could be recovered by confining these cells into synthetic channels of cell-sized dimensions (Wu et al., 2019b, 2020) (Figure 5C). The mere restoration of confinement similar to that imposed by the cell wall thus was able to determine success in segregation, which clearly shows that physics effects are at play, since the biological content of the cells was the same in both shapes.

The entropic spring-like characteristics of nucleoids was experimentally demonstrated *in vivo* and *in vitro* (Pelletier et al., 2012; Wu et al., 2019a,b). Pelletier et al. (2012) revealed that isolated *E. coli* chromosomes would accordingly compress and expand through manipulation with a microchannel-sized piston. The contribution of the entropic forces was tested in *E. coli* cells that contained 1 or 2 chromosomes and had a cell length that was artificially elongated to reach much larger sizes. The cell elongation resulted in the expansion of the chromosomes up to a much longer but finite size (Wu et al., 2019b), indicating that under normal physiological conditions the chromosome acts as a compressed spring. Moreover, two nucleoids distributed to 1/4 and 3/4 positions along the length of the elongated cell (Wu et al., 2019b) (Figure 5D). The cellular positioning was recapitulated in a molecular dynamics simulation as a pressure balance between the nucleoid’s entropic spring compression and the cytosolic molecular crowding (Figure 5E). Widening the *E. coli* cells in all dimensions led to the unfolding of the circular chromosome into a toroidal-shaped chromosome (Wu et al., 2019a). From both studies it appears that relieving cell wall confinement led to occupation of newly vacated cellular space by the nucleoid. All these findings are in accordance with the theoretical framework of the entropic-spring nature of chromosomes under confinement and the accompanying tendency to spontaneously de-mix into two spatially separated polymers.

## Discussion and Outlook

In this review, we described various mechanisms and physical principles that underlie chromosomal segregation in bacteria. Generally, multiple of these mechanisms are simultaneously active within the same organisms to assure a symmetric distribution of replicated genetic material over the daughter cells.

Mixed chromosomes, by their mere physical nature as long circular polymers, will avoid each other and spontaneously move apart to maximize their conformational entropy and minimize the free energy. This entropic de-mixing could very well provide a common primordial driver of segregation throughout taxa. Spontaneous segregation of sisters, with the concomitant inward movement of the yet un-replicated mother chromosome (Jun and Mulder, 2006), elegantly explains the central positioning of the replisome (Lemon and Grossman, 1998, 2000; Mangiameli et al., 2017) and inward movement of loci (Mangiameli et al., 2017) before their replication (Japaridze et al., 2020). Currently, evidence for the contribution of such entropic forces to global segregation of genome-sized DNA polymers has mainly been obtained from simulation studies. Experimental work thus far merely provided indirect evidence of emergent features of such physical models, like the entropic spring-like nature of the nucleoid.

Cells feature a myriad of protein systems that additionally come into play in regulating segregation. For example, SMCs load onto the nucleoid, apparently rather uniformly in *E. coli* (Mäkelä and Sherratt, 2020) or at specific *parS* sequences in *B. subtilis* (Wang et al., 2017) and *C. crescentus* (Tran et al., 2017), to slide along the genome and locally loop it into a bottle brush structure (Petrushenko et al., 2010; Wang et al., 2014a; Lioy et al., 2018). SMCs thus compact the genome as well as interact with topoisomerases that allow them to resolve inter- and intra-chromosomal links. Progressive lengthwise DNA compaction by SMCs may lead to formation of clusters and the amounting mass of replicating DNA may build up stress in the process, which is released by the action of topoisomerase strand-passage activity (Figure 3G). This phenomenon may underlie what was reported as waves of clustered chromosomes and consequent segregation described by Fisher et al. (2013). As an ultimate result of the continued DNA condensation, SMC axial cores (Mäkelä and Sherratt, 2020) form on the individualized chromosomes. Repulsion between the emanating loops also drives apart the untangled sisters under cylindrical confinement (Brahmachari and Marko, 2019). This exemplifies how spontaneous de-mixing of genome-sized polymers can synergistically be catalyzed by the local action of proteins to segregate replicated chromosomes during their individual global organization.

Proteinaceous mechanisms also contribute to global chromosome segregation. In *B. subtilis* and *C. crescentus*, ori-proximal *parS* functions as a handle on sister chromosomes for being pulled apart by the *parABS* system. These combined actions–sister-selective compaction by SMCs and the pulling mechanism by *parABS*–could instantiate repulsion between sisters upon individualization directly after replication initiation.

These multiple contributors to segregation are at play in most bacteria and appear to be well conserved. An exception is the *parABS* system, which is beneficial for segregation, but not essential, as it is lacking in *E. coli*, but clearly contributes to chromosome segregation and loading of SMCs onto chromosomes in *B. subtilis* and *C. crescentus*. Multi-component mechanism such as *parABS*, SMCs, or combinations of both, may have evolved to ensure segregation in increasingly large and more complex organisms with increasing genome sizes. The presence and conservation of SMC structure and function throughout all kingdoms of life (Cobbe and Heck, 2004) strongly suggest that it emerged very early in evolution. Analogously, the absence of *parABS* in *E. coli* and the versatile functions of the system’s homologs in bacterial species (Jalal and Le, 2020) indicate that its role in segregation has developed later in some prokaryotes. It is tempting to hypothesize that spontaneous de-mixing was evolutionarily the earliest form of DNA segregation, as it is the simplest physical mechanism.

New model organisms, beyond the well-studied trio of *E. coli*, *C. crescentus*, *or B. subtilis*, may provide new insights as well. One can for example study the effects of cell shape by looking at non-rod-shape bacteria such as *Staphylococcus* which are spherical, yet provide reliable segregation. In search of a model organisms for primordial segregation, cell-wall-less bacteria such as *Mycoplasma pneumonia* are also of interest because of its very small genome (Himmelreich et al., 1996; Hutchison et al., 2016).

Thus far, it has remained difficult to disentangle the relative importance of various individual mechanisms in segregating genome-sized substrates in cells. To investigate this in an alternative way, a recently proposed bottom-up approach named “genome-in-a-box” may provide an interesting starting-point (Birnie and Dekker, 2020). Here, a Mbp-long genome, stripped of all proteins is confined within a microfluidic device, and single components such as SMCs, *parABS*, or other genome-structuring proteins can be added to monitor their individual effects, irrespective of the additional mechanisms that are normally simultaneously at play in cells. Microfluidics-based droplet (oil in water) or liposome techniques allow, in a very controllable way, to generate vesicles of various size and composition (Wu and Dekker, 2016; Deshpande and Dekker, 2019). These can encapsulate bacterial chromosomes and thus provide a model test object to study the dynamics of genome-sized polymers under confinement. By mixing two such minimal genomes, one may be able to investigate how confinement and the contributions of different proteins prime and influence the segregation process. While various technical challenges arise in trying to establish this genome-in-a-box methodology, a bottom-up technology such as this has great potential for providing essential insights in the mechanisms underlying DNA segregation.

Segregation and its coordination with replication and division undeniably belongs to the fundamental processes that sustain all life forms. Understanding how replicated chromosomes in the simpler organisms are driven to disentangle and partition into daughter cells lays the groundwork for understanding the mechanism of chromosome segregation in more complex organisms like eukaryotes. Unraveling the basic principles and contributions of different mechanisms may furthermore lead to applications in ultimately creating the first synthetic cell (Schwille et al., 2018; Spoelstra et al., 2018; Deshpande and Dekker, 2019; Gaut and Adamala, 2021).

## Author Contributions

CG and AJ contributed equally to writing the review. All authors wrote the manuscript and CD supervised the project.

## Conflict of Interest

The authors declare that the research was conducted in the absence of any commercial or financial relationships that could be construed as a potential conflict of interest.
